# Systematics Review and Phylogeny of Cyrtophyllitinae Zeuner, 1935 *sensu* Gorochov, Jarzembowski & Coram, 2006 (Ensifera, Haglidae), with Description of Two New Species †

**DOI:** 10.3390/insects15060396

**Published:** 2024-05-29

**Authors:** Jun-Jie Gu, Wei Yuan, Rong Huang, Dong Ren, Hong-Xing Chen

**Affiliations:** 1 College of Agronomy, Sichuan Agricultural University, Chengdu 611130, China; 2 College of Life Sciences, Capital Normal University, 105 Xisanhuanbeilu, Haidian District, Beijing 100048, China; rendong@mail.cnu.edu.cn

**Keywords:** Archaboilinae subfam. nov., fossil, Hagloidea, Middle Jurassic, wing venation

## Abstract

**Simple Summary:**

The systematics of Cyrtophyllitinae Zeuner, 1935 *sensu* Gorochov, Jarzembowski & Coram, 2006 was revised. A new subfamily Archaboilinae subfam. nov. and a new genus, *Pararchaboilus* gen. nov. were erected. Two new species, *Archaboilus ornatus* sp. nov. and *Vitimoilus gigantus* sp. nov., are described from the Middle Jurassic China.

**Abstract:**

A phylogeny of Cyrtophyllitinae Zeuner, 1935 *sensu* Gorochov, Jarzembowski & Coram, 2006, based on wing morphology, is presented including all genera. Cyrtophillitinae is found to be paraphyletic. Except for *Cyrtophyllites rogeri* Oppenheim, 1888, all other species were moved from the subfamily Cyrtophyllitinae (Hagloidea, Haglidae). Consequently, a new subfamily Archaboilinae subfam. nov. was erected and accommodates most of the previous cyrtophillitine taxa, except *Cyrtophyllites rogeri*. The type genus *Archaboilus* Martynov, 1937 of the new subfamily was designated; a new genus, *Pararchaboilus* gen. nov., was erected with the designation of type species *Pararchaboilus cretaceus* comb. nov. From the Middle Jurassic deposits of China, two new species, *Archaboilus ornatus* sp. nov. and *Vitimoilus gigantus* sp. nov., are described.

## 1. Introduction

As a main constituent family of the superfamily Hagloidea, the Haglidae existed from the Upper Permian to the Early Cretaceous and were most abundant from the Triassic to the Middle Jurassic [1,2,3]. The species of this family were plentiful and diverse—classified into nine subfamilies: Haglopterinae Gorochov, 1986; Haglinae Handlirsch, 1906; Isfaropterinae Martynov, 1937; Triassaginae Gorochov & Maehr, 2008; Tshorkuphlebiinae Martynov, 1937; Voliopinae Gorochov, 1986; Bachariinae Gorochov, 1988, Angarohaglinae Gorochov, 1995 and Cyrtophyllitinae Zeuner, 1935 [4,5,6,7,8,9,10]. However, when compared to the family Prophalangopsidae, haglid insects were not as diverse in the Mesozoic of China. Only a few species are definitely assigned to Haglidae with corresponding subfamily assignments [11,12,13,14,15].

The subfamily Cyrtophyllitinae was erected by Zeuner in 1935, and originally included the genera *Procyrtophyllites* Zeuner, 1935 and *Cyrtophyllites* Oppenheim, 1988 [10,16]. Gorochov added *Archaboilus* Martynov, 1937, *Tasgorosailus* Gorochov, 1990 and *Protohagla* Zeuner, 1962 to this subfamily [8,17,18]. Later, Gorochov et al. revised this subfamily, adding *Vitimoilus* Gorochov, 1996 and removing *Protohagla* Zeuner, 1962 [10,19].

Cyrtophyllitinae is characterized by the presence of a vein interpreted as a ScA (a secondary tegminal C) running along the anterior wing margin, the presence of an oblique vein connecting the base of RP (RS) and MA (2MA_1_), and a strong CuPaβ (CuA_2_) [10]. Currently, there are 11 species from four genera assigned to Cyrtophyllitinae recorded from the Early Jurassic to Early Cretaceous [2,10,12,13,14]. *Archaboilus* is the most diverse genus of Cyrtophyllitinae, including six species recorded from the Lower Jurassic (Kyrgyzstan and Russia) to the Middle Jurassic (Yanliao Biota of China). However, the assignment of *A. polyneurus* Gu, Yue & Ren, 2021 to the genus was questioned by Gorochov and Coram [2] because its base of the MP and free CuA are situated further from each other than other *Archaboilus* species. 

The main objective of this study was to investigate the phylogeny and systematics of Cyrtophyllitinae *sensu* Gorochov Jarzembowski & Coram, 2006 based on the wing venation characters. In addition, two new species from the Yanliao Biota of China (*Archaboilus ornatus* sp. nov. and *Vitimoilus gigantus* sp. nov.) are described.

## 2. Materials and Methods

### 2.1. Materials Examined and Terminology

All collections described in this contribution were examined with an OLYMPUS SZX16 dissecting microscope (Olympus, Tokyo, Japan). The photographs were taken using a Canon EOS 550D digital camera coupled to a Canon 50 mm macro lens (Canon, Tokyo, Japan). The specimens studied here were collected from the Middle Jurassic Jiulongshan Formation of Daohugou Village, Ningcheng city, Nei Mongol Autonomous Region (Inner Mongolia), China [20,21,22] and are housed at the Key Laboratory of Insect Evolution & Environmental Changes, Capital Normal University (CNU), Beijing, China. 

The wing venation nomenclature used in this paper is based on the interpretation of venation by Béthoux and Nel [23,24]. Corresponding abbreviations are: ScA, anterior subcosta; ScP, posterior subcosta; RA, anterior radius; RP, posterior radius; MA, anterior media; MP, posterior media; CuA, anterior cubitus; CuP, posterior cubitus; CuPaα, the anterior branch of the first posterior cubitus; CuPaβ, the posterior branch of the first posterior cubitus; and CuPb, the second posterior cubitus. The term ‘handle’ describes a strong cross vein appearing as a main vein.

### 2.2. Phylogenetic Analyses

Because of poor preservation and insufficient illustration, the species *Archaboilus kisylkiensis* Martynov, 1937 and *A. similis* Zherikhin, 1985 are excluded from this phylogenetic analysis [25]. All remaining species from the four genera constitute the ingroup, covering 85% of all Cyrtophyllitinae species (Table 1). The monophyly of Hagloidea and Haglidae was debated [24]. We choose *Euhagla saurensis* Gorochov, 1986, a well-recorded species from the most diverse subfamily Haglinae of Haglidae, as one of the outgroups. *Liassophyllum caii* Gu, Qiao & Ren, 2012, a well-known species from family Tuphellidae of Hagloidea, was selected as another outgroup for this study [26]. This species is recorded from the Middle Jurassic and exhibits some similarities of wing venation with the type species of Cyrtophyllitinae.

A total of 24 forewing characters were selected by reviewing the obtained specimens and descriptions in the relevant literature (Table 2). Some characters were derived from the study conducted by Béthoux and Nel [24], although certain character states were modified. All characters are unordered and carry equal weight. Missing data were coded with a question mark (?). The data matrix used for the phylogenetic analysis is shown in Table 3.

The above characters matrix was analyzed using the phylogenetic analysis software WinClada v.1.61 [27]. The tree search was executed using a heuristic search method with the following settings: 10,000 maximum trees to keep, 1000 replications, 100 starting tree replications, and a multiple TBR + TBR search strategy. In addition, we also searched for the most parsimonious tree using the software TNT v.1.5 [28]. To evaluate the tree support, a nonparametric bootstrap analysis was performed with 1000 replicates. The final trees were visualized using WinClada v.1.61 [27].

**Figure 1:**
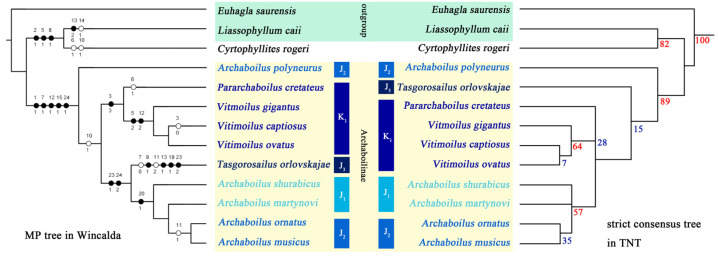
The most parsimonious tree from Winclada and strict consensus tree from TNT. Numbers above the branches in the tree obtained by WinClada indicate the number of characters; white circles indicate homoplastic characters, and black circles indicate non-homoplastic characters. Bootstrap values are annotated below each branch of the tree by TNT. Colored names indicate different geologic ages.

**Figure 2:**
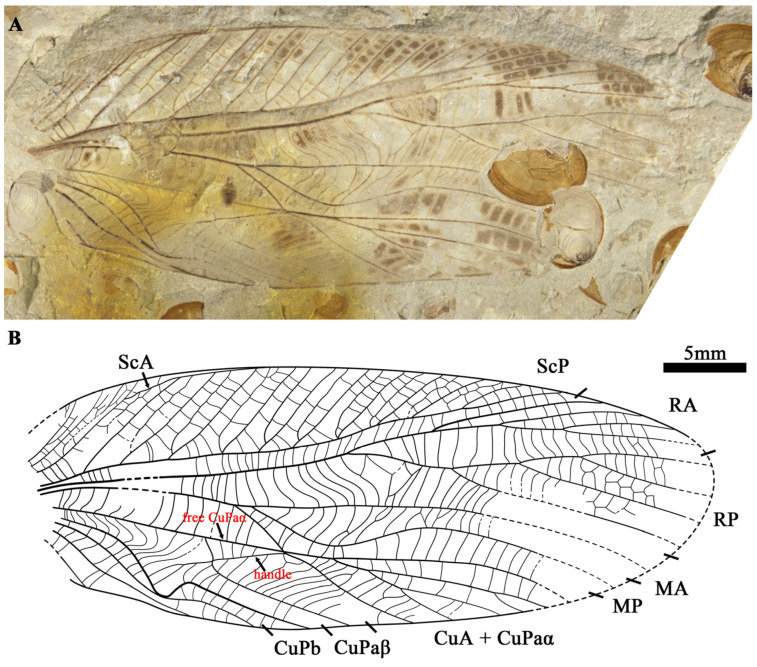
*Archaboilus ornatus* Gu, Ren et Chen sp. nov., holotype, CNU-ORT-NN2011033, male. (**A**) Photo of wing. (**B**) Line drawing. All to the same scale bar: 5 mm.

**Figure 3:**
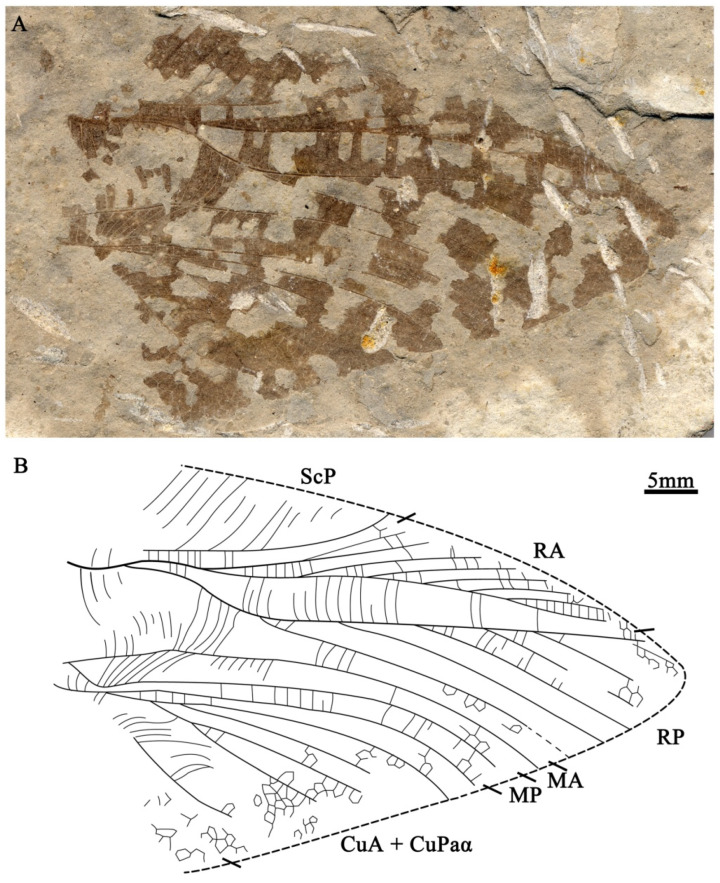
*Vitimoilus gigantus* Gu, Ren et Chen, sp. nov., holotype, CNU-ORT-LJ2009009, male. (**A**) Photo of wing. (**B**) Line drawing. All to the same scale bar: 5 mm.

## 3. Results

### 3.1. Phylogenetic Results

Winclada (with the NONA program) found one most parsimonious tree (MPT) (length = 42; consistency index = 0.80; retention index = 0.81); TNT calculated two MPTs. Character states were mapped on the strict consensus tree using WinClada ver.1.61 [27].

Overall, the results obtained from both analysis methods are similar. *Cyrtophyllites rogeri* Oppenheim, 1888 forms a clade with one of the outgroups, *Liassophyllum caii*, supported by characters 2, 5 and 8. The other species of Cyrtophyllitinae *sensu* Gorochov, Jarzembowski & Coram, 2006 formed a monophylum supported by several synapomorphies (chars. 1:1, 7:1, 12:1, 15:1, 24:1), with a high supported value. *Archaboilus polyneurus* appears to be the basal position of the clade. The clade consisting of *Archaboilus*, *Vitimoilus*, *Pararchaboilus cretaceus* comb. nov. (=*Cyrtophyllites cretaceus*) and *Tasgorosailus orlovskajae* has a very low support value but is resolved as a sister to *A. polyneurus*. The relationships within this clade show slight differences between the two analyses. *Vitimoilus* is well-confirmed as a monophyletic group and forms a clade with *P. cretaceus* comb. nov., supported by character 3 in the Winclada analysis. In the Winclada analysis, *Tasgorosailus orlovskajae* is discovered as a sister group to the clade of *Archaboilus* species (except *A. polyneurus*), while in the TNT analysis it appears at a more basal position. Except for *A. polyneurus*, all other *Archaboilus* species form a clade by a synapomorphy (char. 20:1).

### 3.2. Systematic Palaeontology

Order: Orthoptera Olivier, 1789
Suborder: Ensifera Chopard, 1920
Superfamily: Hagloidea Handlirsch, 1906
Family: Haglidae Handlirsch, 1906
Subfamily: Archaboilinae Gu, Ren et Chen, subfam. nov.

Diagnosis. This subfamily is characterized by the presence of a distinct ScA crossing the area between the anterior wing margin and ScP. It is different from Prophalangopsidae by its ScA running along the anterior wing margin and not cutting the basal branches of ScP. When compared to other Haglidae subfamilies, R forks into RA and RP, usually close to the middle of the wing; the area between RP and MA usually contains an oblique vein (distinct or weak) separating two sets of cross veins in the area with MA distant to RP; the area between R and MA widens and the widest area at the level of or distal to R forks into RA and RP; the part of MA opposite to RP is located at a distant position from RP.

Type genus. *Archaboilus* Martynov, 1937, herein designated (type species: *Archaboilus kisylkiensis* Martynov, 1937).

Included genera. *Archaboilus* Martynov, 1937 (Lower to Middle Jurassic, Kyrgyzstan, Russia and China), *Pararchaboilus* gen. nov. (Lower Cretaceous, UK), *Tasgorosailus* Gorochov, 1990 (Upper Jurassic or Lower Cretaceous, Kazakhstan), *Vitimoilus* Gorochov, 1996 (Lower Cretaceous, Russia and China).

Etymology. The name of this subfamily originated from the name of the first described and diverse genus of this clade, “*Archaboilus*”.

Remarks. After the morphological comparison and phylogenetic analysis, Cyrtophyllitinae Zeuner, 1935 *sensu* Gorochov, Jarzembowski & Coram, 2006 was revised. We incorporated all known species from Cyrtophillitinae into the new subfamily Archaboilinae, with the exception of the species *Cyrtophyllites rogeri*. Further details are in the discussion section.

Genus: *Archaboilus* Martynov, 1937

Revised diagnosis. This genus can be characterized by the following combination of characters: the presence of an oblique vein connecting the middle of the free RP and MA; a free CuPaα approximately the same length as the free CuA; CuPa forks into CuPaβ and CuPaα at the level of the bow of AA1 after its fusion with CuPb.

Included species. *A. kisylkiensis* Martynov, 1937, *A. shurabicus* Martynov, 1937, *A. similis* Zherikhin, 1985, *A. martynovi* Gorochov, 1988, *A. musicus* Gu, Engel & Ren, 2012b, *Archaboilus ornatus* sp. nov. and probably *A. polyneurus* Gu, Yue & Ren, 2021.

*Archaboilus ornatus* Gu, Ren et Chen, sp. nov. (Figure 2)

Diagnosis. ScA relatively short, reaching the anterior wing margin at the level of the origin of MA; MA not undulate (arched); the oblique vein connecting RP and MA weak and formed by several cross veins partially fused together.

Etymology. From the Latin “*ornatus*”, referring to the gorgeous colored spots on the wings.

Material holotype. CNU-ORT-NN2011033, male.

Locality and horizon. Daohugou Village, Shantou Township, Ningcheng County, Inner Mongolia, China; Jiulongshan Formation, Middle Jurassic.

Description. Forewing oval, estimated length 41.8 mm; the area between the ScA and anterior margin filled by numerous weak veinlets, ScA crossing the subcostal area and reaching the anterior margin at the level of divergence of M, most parts of ScA close to the wing margin and not cutting the branches of ScP; ScP long, reaching the anterior margin at 3/4 of the wing length with a branch of the ScP with a secondary vein between them, formed by two row of cells, regularly disposed; area between ScP and RA narrow, with straight cross veins; R forking into RA and RP at the middle of the wing, branches of RA emitting at almost the level of the branches of RP arising, both RA and RP pectinate with four branches, most of the area between the RP branches covered by regular reticulate cross veins; area between R and MA broad, with an oblique vein dividing the cross veins into two parts, the cross veins between MA and the stem of R curved; free M short, forking into MA and MP soon after separating from M + CuA; MA slightly arched, MP obviously curved toward the posterior margin basally; CuA separates from M + CuA basal of ScA, reaching the anterior wing margin; CuA + CuPaα ramified with six terminals reaching the wing margin; handle vein straight; CuPaβ broken by the handle, the part between the handle and CuPa short and straight, the part between the handle and posterior wing margin strongly oblique; cross veins are apparently curved in the basal area of CuPb and CuPaβ; the middle part of CuPb sharply bent toward the posterior margin; forewing covered with many colored spots.

Remarks. Due to the early divergence of the free CuA, the free M, and small stridulate area, this specimen cannot be assigned to *Vitimoilus* Gorochov, 1996. *Cyrtophyllites* species have a basally strongly curved and branched RP, which is clearly different from *A. ornatus* sp. nov. The MA and MP of *Tasgorosailus* are oblique and parallel, which is different from this new species. This new species shares with other *Archaboilus* species the strongly curved base of MP and a very narrow area between CuPaβ and AA1 after their fusion. Compared to other *Archaboilus* species, it is similar to *A. musicus* Gu, Engel & Ren, 2012 from the same locality in most of the wing venation. It can be separated from the latter by its relatively short ScA and the presence of a weak oblique vein connecting RP and MA rather than a distinct oblique vein. It differs from all other *Archaboilus* species due to its non-undulate MA, relatively short ScA, and a weak oblique vein connected to the base of RP.

Genus: *Vitimoilus* Gorochov, 1996

Revised diagnosis. This genus can be characterized by the following combination characters: forewing broad oval and large size; area between the anterior margin and ScA with numerous weak veinlets; ScP undulated with numerous branches; M forking into MA and MP distally; CuA fused with CuPaα distal of the middle of the wing length; compare to other Archaboilinae species, its R forking distally and RP curved toward the anterior margin, the area between R and MA widened, with the widest part after R forks.

Included species. *V. captiosus* Gorochov, 1996, *V. ovatus* Gu, Tian, Yin, Shi & Ren, 2017, *V. gigantus* sp. nov.

*Vitimoilus gigantus* Gu, Ren et Chen, sp. nov. (Figure 3)

Diagnosis. Forewing long and broad, estimated length of male forewing longer than 100 mm; MA slightly arched, not sigmoidal; MP bends before R forks into RA and RP.

Etymology. From the Latin “*gigantus,*” referring to the large size of its forewing.

Material holotype. CNU-ORT-LJ2009009, male, proximal half of the wing is absent.

Locality and horizon. Jianchang County, Liaoning province, China; Yixian Formation, Lower Cretaceous.

Description. Forewing large size, 59.5 mm preserved, estimated length of single wing ca. 113 mm (based on the proportion of the forewing of *V. ovatus* Gu, Tian, Yin, Shi & Ren, 2017) [14]; estimated maximum width ca. 45 mm. ScP long with a large number of branches, area between ScP and RA rather narrow, with straight cross veins; R forks into RA and RP distal of the divergence of MA and MP; RA pectinate with seven branches, RP pectinate with at least seven (probably with eight) branches, the free part of RP curved toward the anterior margin basally; area between R and MA broad with long and curved cross veins; MA simply arched, not sigmoidal; MP bends toward the posterior wing margin before R forks into RA and RP; CuA + CuPaα with six branches preserved (probably with seven); area between CuA + CuPaα branches covered by straight cross veins basally and reticulate cross veins distally.

Remarks. Although the basal half of the wing is absent, we can assign this specimen to the genus *Vitimoilus* Gorochov, 1996 due to the following characters: forewing broad, R forking into RA and RP distally, base of RP curved toward the anterior margin, MP basally curved and very close to the first branch of CuA + CuPaα. This new species is hard to compare with the type species of the genus, *V. captiosus* Gorochov, 1996, owing to the latter’s radius and most parts of the media and cubitus of the forewing are not preserved, but their forewing size varies dramatically. Compared to *V. ovatus* Gu, Tian, Yin, Shi & Ren, 2017 (also from the Jehol biota), this new species is much larger (over 110 mm vs. 55–73 mm), MA is not sigmoidal, MP bends before R forks into RA and RP. Although the cubital and anal area of the new species are not preserved, the widened area between MA and the base of RP indicates that this specimen is likely a male forewing rather than a female [15]. The possibility that this specimen is a female individual of *V. ovatus* Gu, Tian, Yin, Shi & Ren, 2017 can therefore be excluded. The wing length and width of this new species is calculated based on the proportion of known species of *Vitimoilus*. If this inferred size is roughly correct, the species is currently the largest known Jurassic orthopteran species, despite the fact that it is represented by a male specimen. The females of the species may have been larger if they exhibit dimorphism like common orthopterans. The present specimen is slightly longer than the female of *Aboilus lamina* from the Jurassic, which has a maximum forewing length of 104 mm [29,30].

*Pararchaboilus* Gu, Ren et Chen, gen. nov.

Type species. *Pararchaboilus cretaceus* Gu, Ren et Chen comb. nov., herein designated.

Etymology. From the genus name “*Archaboilus*” in reference to their similar wing venation.

Diagnosis. This genus can be characterized by the following combination characters: its R forks into RA and RP distally, RP basally branched, CuPa forks into CuPaβ and CuPaα at the level of the fusion of CuPb and AA1. 

Included species. *Pararchaboilus cretaceus* comb. nov. (=*Cyrtophyllites cretaceus* syn. nov.).

Remarks. *Cyrtophyllites cretaceus* Gorochov, Jarzembowski & Coram 2006 was originally erected by a very fragmentary specimen of male forewing, which only preserved the anal veins and part of the cubitus veins [10]. Then, a new material of this species was discovered showing complete wing venation [2]. Based on these materials, their wings are quite different from *C. rogeri* Oppenheim, 1888 in the following characters: the area between RA and RP is lancet-like, RP does not redirect anteriorly; R forks into RA and RP close to the middle of the wing rather than distal to 3/5 of the wing length; the widest area between R and MA is at the divergence of R. At the same time, it cannot be attributed to *Vitimoilus* Gorochov, 1996 due to the base of its RP not curving toward the anterior margin, the base of CuPb distant to the base of CuPa, and a much smaller stridulate area. It is similar to *Archaboilus* in wing shape, but different in the absence of a distinct oblique vein connected to MA and the base of RP, and AA1 not strongly curved toward CuPaβ after its fusion with CuPb. *Tasgorosailus orlovskajae* has obliquely straight MA and MP, and a very narrow area between MA and MP, which is very different from *P. cretaceus* comb. nov. Thus, *Pararchaboilus* gen. nov. is erected. As a consequence, the species *Cyrtophyllites cretaceus* Gorochov, Jarzembowski & Coram 2006 is proposed as the type species of this new genus, as *Pararchaboilus cretaceus* comb. nov.

## 4. Discussion

Cyrtophyllitinae *sensu* Gorochov, Jarzembowski & Coram 2006 proved to be paraphyletic in the analysis. *C. rogeri* formed a clade with one of the outgroup taxa, *L. caii* (Tuphellidae), and was separated from all other cyrtophyllitine species. The type specimen of *C. rogeri* is not completely preserved; for example, it is hard to confirm whether it had a developed vein ScA crossing the subcostal area, which is the synapomorphy for the subfamily. But, its distally redirected ScP (toward the anterior margin) (char. 2:1) and the very distal divergence of R with a rectangular area of RA and RP (char. 8:1) are not observed in other Cyrtophyllitinae species, even most haglids. In fact, the characters mentioned above, as well as its basally curved RP forming an approximate right angel to RA, are found in most Tuphellidae species (4; 22). Morphologically, this species is close to Tuphellidae rather than to Haglidae. Due to the incomplete preservation and limited knowledge of *C. rogeri*, we suggest excluding it from the subfamily. Further investigation is required to determine its proper family assignment based on the examination of the type specimen. A monophyletic clade consisting of *Archaboilus* Martynov, 1937, *Tasgorosailus* Gorochov, 1990, *Vitimoilus* Gorochov, 1996 and *Pararchaboilus cretaceus* comb. nov. is supported by a series of synapomorphies with a relatively high support value (Figure 1). This group shares a developed a ScA across the area between ScP and the anterior wing margin (char. 1:1, synapomorphic character of Archaboilinae subfam. nov.), which is much different from the other Haglidae subfamilies. A similar structure can be found in another Hagloidea family Prophalangopsidae, but the ScA of the latter always cuts the branches of the ScP (probably a synapomorphic character of Prophalangopsidae). Furthermore, its R forks into RA and RP close to the middle of the wing (char. 7:1), the widest part of the area between R and MA being located at the forking of R (char. 12:1), and the part of MA opposite to RP located at a distant position from RP (char. 15:1), which are discovered as synapomorphies of this clade. Besides, in this group, the length of the handle is approximately the same or longer than the length of CuPaβ between CuPa and the handle. Therefore, we retain the subfamily rank in Haglidae of this group. As the systematic changes of this group, a new subfamily name should be given for this subfamily-level grouping. As *Archaboilus* Martynov 1937 is the earliest reported genus (Early Jurassic) with the most species diversity (7 known species at present, see Gorochov 1995; Gu et al. 2017, 2021) in this group [8,14,15], we designated *Archaboilus* as the type genus of this subfamily; as a consequence, Archaboilinae subfam. nov. is proposed as the new name of this group. 

However, despite obtaining a clear topology, the results indicate that the phylogenetic relationships within the subfamily have not been well resolved. The support values for some branches are generally low. Many known hagloid species often have poorly preserved or unclear wing vein structures, making it difficult to assess their homology. Nonetheless, we can still glean insights into the systematics and relationships among the genera from the results.

The monophyly of *Archaboilus* has not been well confirmed in the analyses. *A. polyneurus* Gu, Yue & Ren, 2021 appears at the base of the subfamily. All other *Archaboilus* species cluster into a clade supported by the free CuPaα sharing the same length as the free CuA. The generic assignment of *A. polyneurus* Gu, Yue & Ren, 2021 was disputed by Gorochov and Coram [2] due to its long free M and different branching pattern of RA and RP, which differ from other *Archaboilus* species. In fact, all other *Archaboilus* species have a relatively short free M, much shorter than their free CuA. Notably, all specimens of *A. polyneurus* exhibit varying degrees of compression deformation, typically in the longitudinal direction, i.e., compression along the anterior–posterior axis [15]. This may introduce potential biases when comparing relative length relationships of certain structures. However, compared to other species, it is more similar to *Archaboilus* species. It shares common features with other *Archaboilus* species, including a distinct oblique vein connecting MA and the middle of RP, which separates two sets of cross veins between the area of RP and MA. Additionally, AA1 strongly curves toward CuPaβ after fusing with CuPb, forming a very narrow area with CuPaβ. Thus, the position of *A. polyneurus* requires further investigation based on newly well-preserved specimens. 

All *Vitimoilus* species have been found in the Early Cretaceous deposits of Russia and China. This genus shares with another Cretaceous genus *Pararchaboilus* gen. nov. the absence of an oblique vein separating two sets of cross veins in the area between the RP and MA (the only synapomorphic character of the clade consisting of these two genera). Instead, they exhibit a more derived character state where the cross veins in this area become long, curved, and closely spaced [2,14]. However, despite this shared character, they display significant differences in wing shape and the structure of the stridulatory area. This sister relationship between the two genera was rejected by TNT analysis (Figure 1). Therefore, their sister group relationship is questionable. Compared to *Archaboilus*, the stridulatory apparatus area in *Vitimoilus*, which extends from the basal anal vein to the posterior branch of CuA + CuPaα, tends to expand toward the middle of the wing as their forewing broadens. The divergence positions of R, M + CuA, and M tend to shift toward the wing’s apex. 

*Tasgorosailus* is a monotypic genus and appears to be in the basal position of Archaboilinae subfam. nov. in the TNT analysis but resolved as a sister of *Archaboilus* in the Winclada analysis. This genus exhibits unique characters that distinguish it from all other species in the subfamily Archaboilinae. Its MA and MP are not arched and slightly oblique straight, forming a distinct narrow area between the base of MA and MP, and its CuPa forks into CuPaβ and CuPaα at the level of the bow of AA1 after its fusion with CuPb. These characteristics are considered as autapomorphies based on the analysis. However, it shares with *Archaboilus* species the synapomorphic condition in which the length of the handle is more than two times longer than the length of CuPaβ between CuPa and the handle, and CuPa forks into CuPaβ and CuPaα distal to the fusion of CuPb and AA1. These shared characters were resolved as synapomorphies of the clade consisting of *Tasgorosailus* and *Archaboilus*.

## 5. Conclusions

Two new species, examined from the Middle Jurassic Jiulongshan Formation of Nei Mongol and the Yixian Formation of Liaoning, China, were described and assigned to *Archaboilus* and *Vitimoilus*, respectively. *Vitimoilus gigantus* Gu, Ren et Chen, sp. nov. is probably the largest known Jurassic orthopteran species to date. After the morphological comparison and phylogenetic analysis, the systematics of subfamily Cyrtophyllitinae Zeuner, 1935 *sensu* Gorochov, Jarzembowski & Coram, 2006 was re-evaluated. A new subfamily, Archaboilinae subfam. nov., was created to accommodate the taxa previously assigned to the Cyrtophyllitinae (*Archaboilus* Martynov, 1937, *Vitimoilus* Gorochov, 1996, *Tasgorosailus* Gorochov, 1990, and *Pararchaboilus* gen. nov.), with the exception of *Cyrtophyllites* Oppenheim, 1888. 

This is an attempt to test the phylogenetic relationships of fossil ensiferans using wing venation characteristics. The results indicate that such work is helpful in improving the systematic and taxonomic understanding of fossil groups, particularly under the limited availability of features.

## Figures and Tables

**Table 1:** Geological age and distribution of Cyrtophyllitinae Zeuner, 1935 *sensu* Gorochov Jarzembowski & Coram, 2006.

Taxon	Distribution	Age	Reference
*Archaboilus kisylkiensis* Martynov, 1937	Kyrgyzstan	J_1_	[5]
*A. shurabicus* Martynov, 1937	Kyrgyzstan	J_1_	[5]
*A. martynovi* Gorochov, 1988	Kyrgyzstan	J_1_	[7]
*A. similis* Zherikhin, 1985	Russia	J_1_	[25]
*A. musicus* Gu, Engel & Ren, 2012	China	J_2_	[13]
*A. polyneurus* Gu, Yue & Ren, 2021	China	J_2_	[15]
*A. ornatus* sp. nov.	China	J_2_	This study
*Cyrtophyllites rogeri* Oppenheim, 1888	Germany	J_3_	[16]
*Pararchaboilus cretaceus* comb. nov.	England	K_1_	[2,10]
*Tasgorosailus orlovskajae* Gorochov, 1990	Kazakhstan	J_3_	[17]
*Vitimoilus captiosus* Gorochov, 1996	Russia	K_1_	[19]
*V. ovatus* Gu, Tian, Yin, Shi & Ren, 2017	China	K_1_	[14]
*V. gigantus* sp. nov.	China	K_1_	This study

Note: J_1_: Early Jurassic, J_2_: Middle Jurassic, J_3_: Late Jurassic, K_1_: Early Cretaceous.

**Table 2:** Definition of morphological characters and states.

No.	Morphological Characters and States
1	ScA crosses the subcostal area: 0, no; 1, yes.
2	ScP redirected anterior margin near the tegmen apex: 0, no (Figure 2); 1, yes (Figure 1, [26]).
3	The area between the RP and MA has a distinct oblique vein separating two sets of cross veins: 0, no (Figure 288, [8]); 1, yes, the oblique vein connected to the middle of the free RP (Figure 1D, [13]); 2, yes, the oblique vein connected to the base of the free RP (Figure 1D, [13]); 3, without a distinct oblique vein but the presence of several long and curved cross veins (Figure 1, [14]).
4	Base of the RP far away from the base of the MA: 0, no, close to each other; 1, yes.
5	RP: 0, curved toward the posterior margin (Figure 1D, [13]): 1, basally curved toward the posterior margin, then redirected to the anterior wing margin (Figure 1, [26]); 2, curved toward the anterior margin (Figure 3, [14]).
6	RP branched: 0, distally (Figure 2); 1, basally (Figure 3A, [2])
7	R forks into RA and RP: 0, distal of 3/5 of the wing (Figure 1, [26]); 1, closer to the middle than to 3/5 of the wing (Figure 2).
8	Area between RA and RP: 0, lancet-like; 1, rectangular (Figure 1, [26]).
9	MP basally curved: no (Figure 3, [17]); 0, 1, yes.
10	Free CuA three times in length longer than free M: 0, no; 1, yes (Figure 2).
11	MA: 0, undulate (Figure 1, [26]); 1, arch (Figure 1); 2, obliquely straight (Figure 3, [17]).
12	The widest part of the area between R and MA, located at: 0, at or after the forking of M and before the forking of R (Figure 288, [8]); 1, at the forking of R (Figure 2); 2, after the forking of R (Figure 3, [14]).
13	MP: 0, sigmoidal (its base curved to the posterior wing margin) (Figure 2); 1, straight (Figure 3, [17]); 2, bowed toward the posterior wing margin (Figure 1, [26]).
14	CuA separated from M + CuA: 0, close to the 1/3 of the wing length (Figure 2); 1, distal to the 2/5 of the wing length.
15	The part of MA opposite RP: 0, bowed toward RP and closely positioned (Figure 1, [26]); 1, located at a distant position from RP (Figure 2).
16	CuA fused CuPaα: 0, basal half of the wing; 1, distal half of the wing (Figure 3, [14]).
17	Cross veins between the CuPb and CuPaβ are strongly curved in the basal part: 0, strongly curved (Figure 2); 1, straight (Figure 3, [14]).
18	M forks into MA and MP: 0, basal or at the level of 2/5; 1, at the level or distal to the 1/2 of wing length (Figure 3, [14]).
19	M + CuA diverges: 0, closer to the one third of the wing length than to the second fifth (Figure 2); 1, closer to the second fifth (Figure 3, [17]); 2, closer to the middle of the wing than to the second fifth (Figure 3, [14]).
20	Free CuPaα vs. free CuA: 0, longer than the free CuA; 1, approximate in length (Figure 2).
21	Basal area between CuPb and CuPa: 0, approximately the same width as the area between the CuPa and the M + CuA; 1, distinctly narrower than the area between the CuPa and M + CuA (Figure 3, [14]).
22	Handle: 0, shorter than the free CuA (Figure 288, Gorochov, 1995); 1, the same length as the free CuA (Figure 2); 2, distinctly longer than the free CuA (Figure 3, [14]).
23	CuPa forked into CuPaβ and CuPaα: 0, at the level of the fusion of the CuPb and AA1 (Figure 288, [8]); 1, at the level of the bow of the AA1 after its fusion with the CuPb (Figure 2); 2, distal of the bow of AA1 after its fusion with the CuPb (Figure 3, [17]).
24	The length of the handle vs. the length of the CuPaβ between the CuPa and handle: 0, shorter; 1, approximately the same length or not longer than twice (Figure 3, [14]); 2. longer than twice (Figure 2).

**Table 3:** Character matrix of 24 characters for the 13 taxa included in this study.

Taxon/Character	01	02	03	04	05	06	07	08	09	10	11	12	13	14	15	16	17	18	19	20	21	22	23	24
*Euhagla saurensis*	0	0	0	1	0	0	0	0	1	0	0	0	0	0	0	0	0	0	0	0	0	0	0	0
*Liassophyllum caii*	0	1	2	1	1	0	0	1	1	0	0	0	2	1	0	0	0	0	0	0	0	1	?	0
*Archaboilus shurabicus*	1	0	1	1	0	0	1	0	1	1	0	1	0	0	1	0	0	0	0	1	0	1	1	2
*Archaboilus martynovi*	1	0	1	1	0	0	1	0	1	1	0	1	0	0	1	0	0	0	0	1	0	1	1	2
*Archaboilus musicus*	1	0	1	1	0	0	1	0	1	1	1	1	0	?	1	0	0	0	0	1	0	1	1	2
*Archaboilus polyneurus*	1	0	1	1	0	0	1	0	1	0	0	1	?	0	1	0	0	0	0	0	0	1	0	1
*Archaboilus ornatus* sp. n.	1	0	1	1	0	0	1	0	1	1	1	1	?	0	1	0	0	0	0	1	0	1	1	2
*Tasgorosailus orlovskajae*	1	0	1	1	0	0	0	0	0	1	2	1	1	?	1	0	0	0	1	0	0	2	2	2
*Pararchaboilus cretaceus*	1	0	3	1	0	1	1	0	1	1	0	1	0	0	1	0	0	0	0	0	0	?	0	?
*Cyrtophyllites rogeri*	?	1	2	1	1	1	0	1	1	1	0	0	0	0	0	0	0	?	0	?	0	?	?	?
*Vitimoilus captiosus*	1	0	0	?	?	?	?	0	1	1	?	?	?	1	?	1	1	1	?	0	1	2	0	1
*Vitimoilus ovatus*	1	0	3	0	2	0	1	0	1	1	2	2	0	1	1	1	1	1	2	0	1	2	0	1
*Vitmoilus gigantus* sp. n.	?	0	3	?	2	0	1	0	1	?	1	2	?	?	1	?	?	?	?	?	?	?	?	?

## Data Availability

No new data were created or analyzed in this study. Data sharing is not applicable to this article.
